# A Simplified SARS-CoV-2 Mouse Model Demonstrates Protection by an Oral Replicon-Based mRNA Vaccine

**DOI:** 10.3389/fimmu.2022.811802

**Published:** 2022-02-16

**Authors:** Vijayakumar Jawalagatti, Perumalraja Kirthika, Chamith Hewawaduge, Ji-Young Park, Myeon-Sik Yang, Byungkwan Oh, Mi Young So, Bumseok Kim, John Hwa Lee

**Affiliations:** ^1^ Department of Veterinary Public Health, College of Veterinary Medicine, Jeonbuk National University, Iksan, South Korea; ^2^ Department of Veterinary Pathology, College of Veterinary Medicine, Jeonbuk National University, Iksan, South Korea

**Keywords:** SARS-CoV-2, mouse model, mRNA, vaccine, oral

## Abstract

A mouse model of SARS-CoV-2 that can be developed in any molecular biology lab with standard facilities will be valuable in evaluating drugs and vaccines. Here we present a simplified SARS-CoV-2 mouse model exploiting the rapid adenoviral purification method. Mice that are sensitive to SARS-CoV-2 infection were generated by transducing human angiotensin-converting enzyme 2 (hACE2) by an adenovirus. The expression kinetics of the hACE2 in transduced mice were assessed by immunohistochemistry, RT-PCR, and qPCR. Further, the ability of the hACE2 to support viral replication was determined *in vitro* and *in vivo*. The hACE2 expression in the lungs of mice was observed for at least nine days after transduction. The murine macrophages expressing hACE2 supported viral replication with detection of high viral titers. Next, *in vivo* studies were carried out to determine viral replication and lung disease following SARS-CoV-2 challenge. The model supported viral replication, and the challenged mouse developed lung disease characteristic of moderate interstitial pneumonia. Further, we illustrated the utility of the system by demonstrating protection using an oral mRNA vaccine. The multicistronic vaccine design enabled by the viral self-cleaving peptides targets receptor binding domain (RBD), heptad repeat domain (HR), membrane glycoprotein (M) and epitopes of nsp13 of parental SARS-CoV-2. Further, *Salmonella* and Semliki Forest virus replicon were exploited, respectively, for gene delivery and mRNA expression. We recorded potent cross-protective neutralizing antibodies in immunized mice against the SARS-CoV-2 delta variant. The vaccine protected the mice against viral replication and SARS-CoV-2-induced weight loss and lung pathology. The findings support the suitability of the model for preclinical evaluation of anti-SARS-CoV-2 therapies and vaccines. In addition, the findings provide novel insights into mRNA vaccine design against infectious diseases not limiting to SARS-CoV-2.

## Introduction

The pandemic, COVID-19, caused by severe acute respiratory syndrome coronavirus 2 (SARS-CoV-2), has resulted in the rapid deployment of prophylactics and therapeutics at a scale never witnessed before (1–4). The rapid development was enabled partly by generation of mouse models of SARS-CoV-2 from prior knowledge derived during the SARS-CoV-1 outbreak (5–7). Laboratory strains of mice are insensitive to SARS-CoV-2 as the mouse homologue of angiotensin-converting enzyme 2 (ACE2) does not support viral replication. The virus uses human ACE2 (hACE2) as a receptor to gain entry into the host cells, and mice expressing hACE2 are highly susceptible to SARS-CoV-1 and develop lung disease (8, 9). Using this prior knowledge, several groups independently developed a SARS-CoV-2 mouse model either by transiently expressing hACE2 [ref (5, 6)] or by generating hACE2 transgenic mice (7, 10). However, these models are not widely available to test drugs and vaccines against SARS-CoV-2, and they require sophisticated laboratory equipment which are not readily available in most labs. Therefore, to generate a mouse model of SARS-CoV-2, we exploited a simplified adenoviral purification protocol that can be performed in any molecular biology lab (11). Recombinant adenoviruses and adeno-associated viruses (AAV) have found wider application in heterologous gene transfer studies (12–15) and have a proven record of safety (16, 17). Here we developed an AAV-hACE2 mouse model and evaluated the feasibility of the approach by testing the efficacy of an mRNA vaccine.

We previously developed a multicistronic self-replicating mRNA vaccine candidate against SARS-CoV-2 by exploiting the Semliki Forest Virus replicon (18). To enable oral delivery of the mRNA vaccine, we exploited the bacteria-mediated gene transfer. The vaccine was safe and induced potent humoral and cellular immune responses. The vaccine protected the hamsters against virus-induced pneumonia (Jawalagatti et al., unpublished). The multicistronic vaccine was designed with an aim to achieve improved protection against the variants. Therefore, as a next step forward, we studied the efficacy of the vaccine in mice in a challenge experiment against delta variant. Moreover, a simplified SARS-CoV-2 mouse model that can be developed in any molecular biology is highly desired. Exploitation of simple and rapid adenoviral purification protocol enabled the development of simplified SARS-CoV-2 mouse model. The vaccine based on parental SARS-CoV-2 gene sequences protected mice against the delta variant challenge. The use of AAV-hACE2 supported viral replication, and the model is highly suitable for small-scale preclinical studies to test drugs and vaccines.

## Materials and Methods

### Ethics Statement

Specific pathogen-free Balb/c mice of 5-week old were procured from Koatech, South Korea. Mice were given standard chow diet and clean water with 12 h light-dark cycle. Jeonbuk National University Animal Ethics Committee approved the experiments (JBNU 2021-027). SARS-CoV-2 was handled in highly secured biosafety labs of Korea Zoonosis Research Institute.

### Cells and Viruses

HEK293T and Vero E6 cell lines procured from ATCC were cultured in DMEM (Lonza, Switzerland) added with 10% FBS (Gibco, USA) and1x penicillin-streptomycin (Gibco, USA) at 37°C in 5% CO_2_. The virus was obtained from Korean Centre for Disease control (KCDC). Vero E6 was used to propagate the SARS-CoV-2 parental strain (BetaCoV/Korea/KCDC/03/2020) and B.1.617.2 delta variant (hCoV/Korea/KDCA119861/2021). Viral titer was determined by plaque assay and stored at −80°C until further use.

### AAV-hACE2 Mouse Model

The adenoviral construct expressing hACE2 (pAAV-hACE2) was created using the AAVpro^®^ helper free system (Takara, Japan). A schematic of the protocol is presented in **Supplementary Figure 1**. The full-length hACE2 gene was amplified from cDNA prepared from Caco-2 cells (**Supplementary Figure 1A**). The hACE2 was cloned into pAAV following directional cloning using Sal I and Xba I restriction enzymes (RE). The positive clones in DH5α *E. coli* were confirmed by colony PCR (**Supplementary Figure 1B**) and RE digestion (**Supplementary Figure 1C**). The HEK293T cells were co-transfected in an equimolar ratio with pAAV-hACE2, pHelper, and pRC2-mi342 plasmids for virus production. The complete transfection, virus isolation, and purification protocol have been described elsewhere (11). The simplified protocol allows purification of virus for small-scale studies in a regular benchtop laboratory. The presence of hACE2 in recombinant AAV2 was confirmed by amplifying full-length hACE2 by PCR following capsid lysis (**Supplementary Figure 1D**). Further, the purified virus was quantified by qRT-PCR (19). The mice were administered intranasally with 2.5×10^8^ genomic equivalents (GE) AAV-hACE2 in 10 µL volume under Avertine anesthesia. The lung samples were collected at days 3, 5, 7, and 9 post-inoculation, and the kinetics of hACE2 expression was determined by qRT-PCR using hACE2 primers and immunohistochemistry (IHC). IHC was carried out using ACE2 rabbit antibody at 1:2000 dilution (Cat. No. 10108-T24, Sino Biological, China). Further, the tissue sections were stained using H & E to assess the impact of AAV-hACE2 on lung histology.

### Vaccine Candidate

The list of bacterial strains, plasmids, and primers used in the present investigation have been listed in **Table 1**. We previously reported the design and construction of the vaccine candidate (18). The vaccine was designed based on the SFV replicon and targets SARS-CoV-2 receptor biding domain (RBD), heptad repeat domain (HR), membrane glycoprotein (M), and epitopes of nsp13. Exploitation of the SFV replicon allows expression of the vaccine construct as a self-replicating mRNA. Further, live-attenuated *Salmonella* Typhimurium (ST) with the genotype Δ*lon, ΔcpxR, ΔrfaL, ΔpagL::lpxE*, and Δ*asd* was used for vaccine delivery. The resultant ST carrying the vaccine construct was designated as JOL3014. To prepare the immunization inoculum, JOL3014 was grown overnight in LB broth and subcultured the following day in 10mL LB broth. Bacteria in the logarithmic phase were pelleted and washed twice in 50mL PBS. The pellet was resuspended in a volume of 2mL. The bacterial number was enumerated based on the OD600 value and used to immunize the mice. The inoculum prepared was used fresh.

**Table 1:** List of bacterial strains, plasmids and primers used in the present study.

Bacteria/Plasmid	Genotypic characteristics	Reference
***S*. Typhimurium**		
JOL3000	Δ*lon* Δc*pxR* Δ*rfaL* Δ*pagL::lpxE* Δ*asd*	Lab stock
JOL3014	JOL3000 carrying pJHL204-V-P2A	(18)
JOL3015	JOL3000 carrying pJHL204	(18)
***E. coli***		
*E.coli*232	F− λ− φ80 Δ(lacZYA-argF) endA1 recA1 hadR17 deoR thi-1 glnV44 gyrA96 relA1 ΔasdA4	Lab stock
JOL3013	*E. coli* 232 carrying pJHL204-V-P2A	(18)
**Plasmids**		
pSFV3-lacZ	amp^R^,SP6 promoter, pBR322 ori	Addgene, USA
pJHL204	asd+, CMV promoter, SV40 promoter, pBR322 ori	Lab stock
pAAV-CMV	Contains a promoter for gene expression, two ITRs and a site for cloning a gene of interest, amp^R^	Takara, Japan
pHelper	Expresses adenovirus E2A, E4, and VA, amp^R^	Takara, Japan
pRC2-mi342	Expresses the Rep and Cap genes of AAV2. Also, expresses hsa-miR-342, a human microRNA that increases AAV2 titer in vector preparation systems, amp^R^	Takara, Japan
**Construct primer**		
V-P2A	Forward - GGGCCCGCCACCATGAGAGTCReverse - GGCGCGCCTTATATTTGTGGCCTG	(18)
**hACE2 primer**	Forward- TCTAGAATGTCAAGCTCTTCCTGReverse- GTCGACCTAAAAGGAGGTCTGAAC	This study
**qRT-PCR primers**		
SARS-CoV-2 N gene	Forward- CACATTGGCACCCGCAATCReverse- GAGGAACGAGAAGAGGCTTG	(20)
*hACE2*	Forward- TCCATTGGTCTTCTGTCACCCGReverse- AGACCATCCACCTCCACTTCTC	This study
AAV ITR	Forward- GGAACCCCTAGTGATGGAGTTReverse- CGGCCTCAGTGAGCGA	(19)

Underlined are the restriction enzyme sites in hACE2 full-length primers.

### Live Virus Neutralization Assay

The sera samples collected in our previous study (Jawalagatti et al., 2021, unpublished) were used to assess the neutralizing antibody response against the parental and delta variants of SARS-CoV-2 (18). Mice were immunized orally with two doses at two weeks apart and the sera samples were collected at week 3 after final immunization. Briefly, heat-inactivated sera samples were diluted serially and incubated with 50PFU SARS-CoV-2 for 2h at 37°C. The virus-serum mixture was used to infect the Vero E6 cells in a 96-well plate for 1h at 37°C. The plates were incubated for 3 days and observed for development of cytopathic effects (CPE), and viral replication was studied by an immunofluorescence assay (IFA).

### Challenge Study

Mice were immunized twice, two weeks apart, with 1×10^8^ CFU *via* the oral route. The mice were challenged three weeks after the final immunization. Four days before the challenge dose, mice were administered intranasally with 2.5×10^8^ genomic equivalents (GE) AAV-hACE2 in 10µL volume under Avertine anesthesia. The mice were challenged with 1×10^4^ PFU of SARS-CoV-2 delta variant in a volume of 10µL *via* the intranasal route under general anesthesia. General anesthesia was achieved by injecting a mixture of 5mg/kg xylazine and 80mg/kg ketamine through the intraperitoneal route. The body weights were monitored on a daily basis, and all animals were sacrificed at day 5 post-challenge. Blood, sera, and lung samples were collected.

### Viral Burden and hACE2 Expression

The viral burden in the lung samples was determined by plaque assay and qRT-PCR (20, 21). The lung samples were weighed and homogenized in DMEM or Trizol for plaque and qRT-PCR assays, respectively. Lung homogenates were clarified by centrifugation at 13000 rpm for 10 min and stored at -80°C. RNA was extracted (GeneAll, South Korea), and cDNA synthesized. The *hACE2* expression was quantified using the gene-specific primers listed in **Table 1**.

### Immunohistochemistry

Formalin-fixed, paraffin-embedded (FFPE) lung sections were deparaffinized by immersing in two changes of xylene, followed by one immersion in xylene-alcohol (1:1 ratio) for 3 min each. The sections were rehydrated by immersing in a series of alcohol gradients of 100%, 95%, 75%, and 50% for 3 min each. The sections were washed in tap water and incubated in 3% H_2_O_2_ in methanol for 10 min to block endogenous peroxidase activity. Following a PBS wash, antigen retrieval was performed by incubating at 100°C for 30 min in 0.5mM citrate buffer pH 6.0. Blocking was performed using 3% BSA at RT for 2 h. The sections were incubated with ACE2 antibody at 1:2000 (Cat. No. 10108-T24, Sino Biological, China) dilutions overnight at 4°C. Following a PBS wash, goat anti-rabbit IgG HRP was added at 1:5000 dilutions and incubated at RT for 1h. The sections were developed using DAB substrate.

### Histopathology

Lung sections were subjected to histopathological analysis following H & E staining.

### Statistical Analysis

Data was analyzed using GraphPad Prism 9.0 and IBM SPSS^®^. Details of the test used and number of animals are indicated in the figure legends. A p-value of <0.05 was considered statistically significant.

## Results

### hACE2 Mouse Model

**Figure 1:**
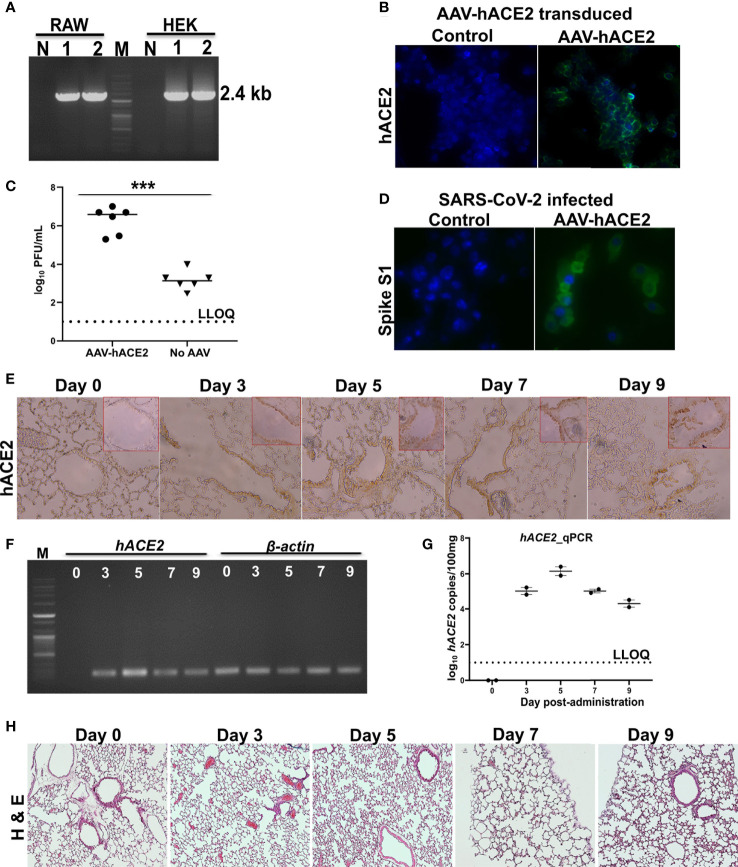
Expression kinetics of hACE2. **(A)** *hACE2* mRNA expression in AAV-hACE2 transduced HEK and RAW cells by RT-PCR. Lane M- DNA molecular weight marker; Lane 1, 2- replicates showing the amplification; Lane N- absence of amplification in non-transduced cells. **(B)** Expression of hACE2 in AAV-hACE2 transduced RAW cells by IFA using ACE2 antibody. Bright green fluorescence indicating the expression of hACE2 on cell surface. AAV-hACE2 transduced RAW cells were infected with SARS-CoV-2 at 0.1 moi and viral replication was determined by **(C)** plaque assay and **(D)** IFA using spike S1 antibody. Mice were intranasally transduced with AAV-hACE2 and expression of hACE2 in the lungs was evaluated by **(E)** IHC using ACE2 antibody, **(F)** RT-PCR to amplify a fragment of ACE2 and **(G)** qRT-PCR. Lane M- DNA molecular weight marker; Lane 0, 3, 5, 7 and 9 indicate the lung sampling day after transduction. Amplification of internal control β-actin has been shown. **(H)** H & E stained lung tissues after adenoviral transduction. Dashed line in **(C)** and **(G)** represents lower limit of quantification (LLOQ). Data information: Data in **(C)** was analysed by Mann Whitney test. Data in **(E–H)** are representative of two mice at each time point from a total of 10. Data presented as mean ± SEM at 95% CI. ***p < 0.001.

The chosen laboratory mice are non-permissive to SARS-CoV-2 as mouse ACE2 does not support viral binding (6, 22). Therefore, to sensitize the mice to SARS-CoV-2, we transiently transduced a recombinant adeno-associated virus (AAV) encoding hACE2 (AAV-hACE2) into the lungs of Balb/c mice. We evaluated the expression of hACE2 and its ability to support viral replication in RAW cells. The expression of hACE2 was confirmed by RT-PCR (**Figure 1A**) and IFA (**Figure 1B**). Further, we detected higher titers of SARS-CoV-2 in AAV-hACE2-transduced RAW cells than in the control cells by plaque assay (**Figure 1C**). The ability of RAW cells expressing hACE2 to support viral replication was confirmed by spike protein IFA (**Figure 1D**). Next, to study the expression kinetics of hACE2 *in vivo*, six-week-old Balb/c mice were transduced intranasally with 2.5 × 10^8^ AAV-hACE2 genomic equivalents (GE). The hACE2 expression was observed in the epithelial cells and pneumocytes of the lungs of transduced mice (**Figure 1E**). Of note, hACE2 protein expression in lungs was detected on all experimental days. Further, we detected *hACE2* expression in lung samples collected on all days by RT-PCR (**Figure 1F**) and qPCR (**Figure 1G**). To evaluate if AAV-hACE2 administration affected the lungs, we carried out histopathological analysis. The H & E-stained lung sections revealed no retrogressive tissue changes compared to the healthy lung tissue (**Figure 1H**), affirming the safety of AAV-hACE2 administration. The hACE2 expression kinetics, histopathological evaluation, and ability to support viral replication affirmed the suitability of the model to evaluate our vaccine candidate in mice.

### Assessment of Cross-Protective Neutralizing Antibodies Against the Delta Variant

**Figure 2:**
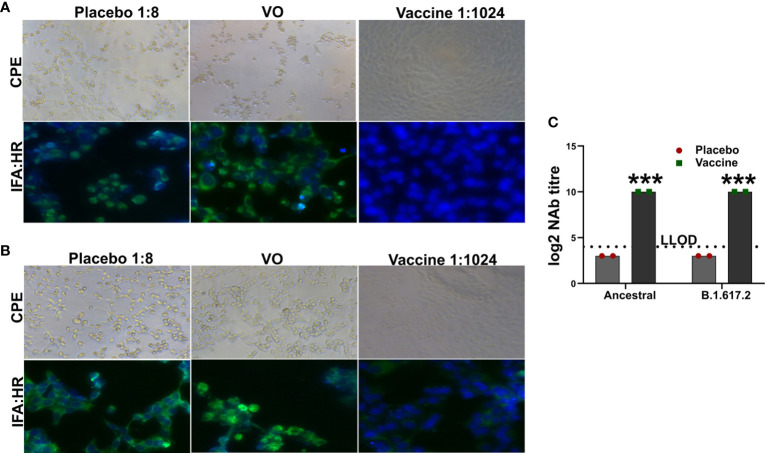
Assessment of neutralizing antibody titer. The inhibition of viral replication by the immune mice sera was evaluated by IFA using HR antibody. IFA images showing neutralization of **(A)** ancestral SARS-CoV-2 and **(B)** B.1.617.2 delta variant. **(C)** The log2 NAb titer has been shown in the right panel. Dashed line represents lower limit of detection (LLOD). Data information: The data was analyzed by two-way ANOVA using Šídák’s multiple comparisons test. Data presented as mean ± SEM at 95% CI. ***p < 0.001.

We created a multicistronic vaccine candidate in an effort to achieve improved protection against SARS-CoV-2 variants. Therefore, we determined the cross-protective neutralizing antibodies in the immune mice sera against delta variant. The sera potently neutralized both the ancestral and B.1.617.2 SARS-CoV-2 with a log2-transformed neutralization antibody (NAb) titer of 10 (**Figures 2A–C**). The NAb titers detected were verified by an IFA assay to study viral replication. Absence of reduction in NAb against the delta variant suggested elicitation of potent cross-protective antibodies by the vaccine.

### Evaluation of Suitability of the Model in an Immunization and Challenge Experiment

**Figure 3:**
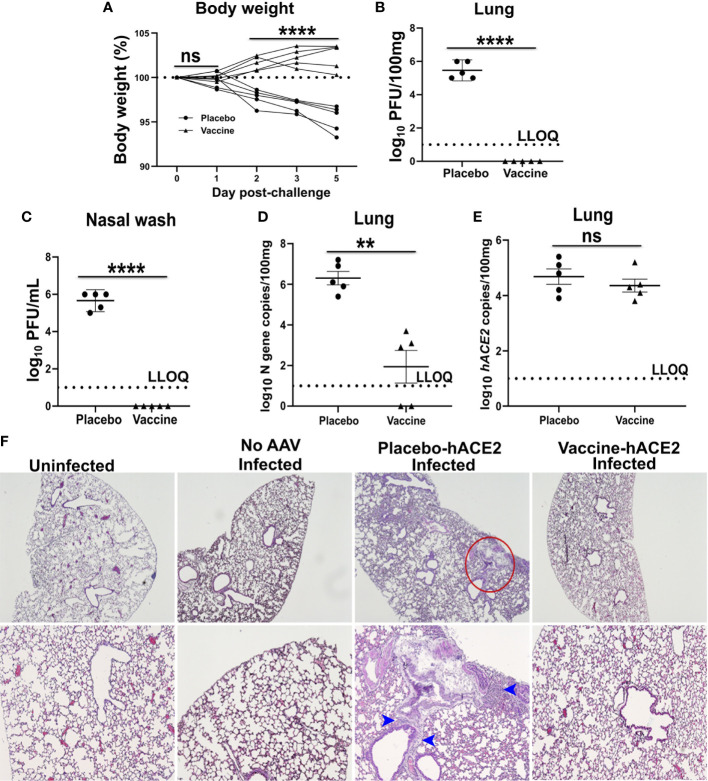
Demonstration of protection by an oral mRNA vaccine candidate. Male mice were immunized with 1 × 10^8^ CFU orally twice at two weeks interval and challenged intranasally with SARS-CoV-2 delta variant three weeks later. Mice were administered AAV-hACE2 4 days before the challenge infection. **(A)** Body weight was monitored for five days following challenge. Each line represents one individual mice. Live virus in **(B)** lung and **(C)** nasal wash was measured by a plaque assay. **(D)** SARS-CoV-2 N gene and **(E)** *hACE2* copies in lung was measured by qRT-PCR. Dashed line in **(B–E)** represents lower limit of quantification (LLOQ). **(F)** H & E stained lung tissue sections. Red circle indicates the area of interstitial pneumonia and blue arrowheads denote the lymphocytic infiltrate. Data information: Protection data was derived from five biologically independent mice per group. Data presented as mean ± SEM at 95% CI. Data in **(A)** was analyzed by two-way ANOVA using Šídák’s multiple comparisons test. Data in **(B–E)** was analysed by Mann Whitney test. **p < 0.01, ****p < 0.0001 and ^ns^p > 0.05.

Next, suitability of the model to support evaluation of a vaccine candidate was studied. For this purpose, mice were immunized with two doses, two weeks apart, of 1×10^8^ CFU *via* the oral route. Mice were challenged at week 3 after the final immunization with an inoculum of 1×10^4^ PFU SARS-CoV-2 delta variant. Four days before the challenge infection, mice were administered intranasally with 2.5 × 10^8^ AAV-hACE2. The mice were sacrificed on day 5 post-challenge, and lung samples were collected. The AAV-hACE2 supported viral replication with detection of viral loads in the lungs and nasal washes in all placebo control mice. A weight loss of 4-7% was observed in the placebo group on day 5 post-challenge (**Figure 3A**). The mean log10-transformed infectious viral loads in the lungs and nasal washes were recorded as 5.86 PFU/100mg (**Figure 3B**) and 6.34 PFU/mL (**Figure 3C**), respectively. Further, mean log10-transformed viral N gene copies in the lungs numbered 6.3/100mg (**Figure 3D**). On the contrary, the immunized mice were protected against weight loss and viral replication. No live virus was detected in the lungs or nasal washes of immunized mice; however, a mean log10-transformed N gene copy number of 1.94/100mg was detected in the lungs, albeit at a low level. The *hACE2* expression was detected in the lungs of both placebo and vaccinated mice (**Figure 3E**). Next, we assessed the histological changes in the lungs of challenged mice following H & E staining. The lesions in the placebo controls were characteristic of mild to moderate interstitial pneumonia with inflammatory cell infiltration, congestion, and hemorrhage (**Figure 3F**). The immunized mice were protected against SARS-CoV-2-induced lung disease. Taken together, the findings support the suitability of the simplified mouse model to evaluate potential anti-SARS-CoV-2 vaccines and therapies.

## Discussion

In the present study, we generated a mouse model by transiently transducing a laboratory mouse strain with AAV-hACE2. We report a simplified SARS-CoV-2 mouse model using AAV encoding the hACE2 that can be constructed in any molecular biology lab. Although the model has the limitation of being suitable only for small-scale studies, it serves as a powerful tool for rapid preclinical testing of drugs and vaccines against SARS-CoV-2. Further, we demonstrate the utility of the model by recording the effectiveness of an oral mRNA vaccine to confer protection against the B.1.617.2 delta variant.

Laboratory strains of mice generally are non-permissive to SARS-CoV-2, as mouse ACE2 does not support viral entry (6, 22). The fact that SARS-CoV uses the hACE receptor for cellular entry was exploited to develop the mouse model of SARS-CoV-2 infection and pathogenesis (5–7). This expedited the development of mouse models of SARS-CoV-2 to test drugs and vaccines (5, 6, 10, 23). However, these models are not widely available, and they require expensive laboratory equipment. Therefore, a mouse model that can be developed in any lab with standard equipment is highly desired. Herein we report a mouse model of SARS-CoV-2 using a simple and rapid adenoviral purification protocol (**Supplementary Figure 1**) (11). We sensitized Balb/c mice to SARS-CoV-2 infection through hACE2 expression in the lungs by adenoviral transduction (**Figure 1**). The AAV-hACE2-transduced mice supported SARS-CoV-2 replication with detection of viral titers in the lungs and nasal washes (**Figures 3A–D**). Further, challenged mice developed lung lesions characteristic of interstitial pneumonia (**Figure 3F**). Viral replication and lung disease in hACE2-expressing mice have been reported previously (5, 6). Similar pathological lesions have been observed in humans and other animals infected with SARS-CoV-2 (24–29). Collectively, our results demonstrate SARS-CoV-2 replication and subsequent development of lung disease in infected mice provided hACE by adenoviral transduction.

Notwithstanding the fact that our model supported viral replication and development of lung disease, the magnitude of viral replication detected was comparatively lower than that observed in other hACE2-adenoviral models (5, 6). Consistent with virological data, mice lost less weight and developed only mild to moderate lung disease (**Figure 3F**). This discrepancy can be attributed to the AAV serotype used in the present investigation, as AAV tissue tropism varies with serotype (30). Our study used serotype 2, whereas other studies have used serotype 5, which might be better suited to deliver genes to lungs (30, 31). Therefore, we recommend the use of AAV serotype 5 or other serotypes such as AAV 1 [ref (32)] and AAV 6 [ref (32)] for future studies.

The COVID-19 pandemic resulted in rapid deployment of vaccines at a scale never witnessed before, with the US Food and Drug Administration (FDA) granting full approval to the two-dose Pfizer-BioNTech vaccine (33). However, due to emerging variants and disproportional vaccine distribution, the global SARS-CoV-2 burden is steadily increasing (34–38). This has necessitated the development of next-generation vaccines to tackle the antigenic diversity. Previously, we developed a *Salmonella*-mediated multicistronic vaccine targeting the RBD, HR, M, and epitopes of nsp13 of SARS-CoV-2 [ref (18)]. The use of bacteria for gene transfer enables oral delivery of the mRNA vaccine, and to our knowledge, our studies are the first to demonstrate an oral mRNA vaccine to combat infectious diseases (39). We previously observed that oral route of immunization elicited potent mucosal IgA response in the lungs (Jawalagatti et. al., unpublished). Moreover, elicitation of mucosal response at respiratory sites following oral immunization has been documented earlier (40). The findings highlight the ability of oral vaccine to induce mucosal response in the respiratory tract. The self-replicating mRNA vaccine design enabled by exploiting the SFV replicon elicited potent humoral and cellular responses in mice and protected hamsters against challenge infection with both the ancestral and delta variants of SARS-CoV-2 (Jawalagatti et. al., unpublished). To further verify the findings and to illustrate the utility of the mouse model, we determined the cross-protective ability of the vaccine in AAV-hACE2-transduced mice challenged with the B.1.617.2 delta variant (**Figure 3**). In agreement with our hamster data, mice immunized orally were protected against viral replication and lung pathology. These results highlight the suitability of our model to test antiviral vaccines and therapies.

In summary, our experiments support the development of a simplified SARS-CoV-2 mouse model. The AAV-hACE2-transduced mice developed lung disease consistent with that of viral replication. Furthermore, the oral mRNA vaccine protected the mice against viral replication and SARS-CoV-2-induced lung disease, demonstrating the feasibility of the approach to test vaccines and drugs against COVID-19.

## Data Availability Statement

The original contributions presented in the study are included in the article/**Supplementary Material**. Further inquiries can be directed to the corresponding author.

## Ethics Statement

The animal study was reviewed and approved by Jeonbuk National University Animal Ethics Committee.

## Author Contributions

VJ and PK designed the study and performed the experiments. VJ analyzed the data, prepared figures and wrote the manuscript. VJ, CH, and J-YP: ABSL3 experiments- viral challenge, sacrifice and sampling. M-SY, BO, MS, and BK: Histopathology. BK provided resources for histopathology experiment. JL: project supervision, acquired funding, commented on the manuscript. All authors approved the final version of the manuscript.

## Funding

This research was supported by Basic Science Research Program through the National Research Foundation of Korea (NRF) funded by the Ministry of Education (2019R1A6A1A03033084).

## Conflict of Interest

The authors declare that the research was conducted in the absence of any commercial or financial relationships that could be construed as a potential conflict of interest.

## Publisher’s Note

All claims expressed in this article are solely those of the authors and do not necessarily represent those of their affiliated organizations, or those of the publisher, the editors and the reviewers. Any product that may be evaluated in this article, or claim that may be made by its manufacturer, is not guaranteed or endorsed by the publisher.

## Supplementary Material

Supplementary Figure 1 Schematic representation of steps followed in development of SARS-CoV-2 mouse model. **(A)** PCR amplification of full-length *hACE2* from cDNA prepared from Caco-2 cells. Lane M- DNA molecular weight marker; Lanes 1-4- different cDNA preparations showing positive amplification.**(B)** Colony PCR confirmation of the cloning of *hACE2* into pAAV2 and **(C)** Positive clone was confirmed by RE digestion using XbaI/SalI restriction enzymes. Lane M- DNA molecular weight marker; Lane c1, c2- colonies; Lane i- RE digested plasmid showing the *hACE2* release. **(D)** Confirmation of presence of *hACE2* in the recombinant adenovirus by PCR. Lane M- DNA molecular weight marker; Lanes s1, s2- sample numbers. *The adenoviral production, isolation and purification was done by following protocols described in Negrini et al., 2020 (ref 11).
